# Potential Involvement of IL-17F in Asthma

**DOI:** 10.1155/2014/602846

**Published:** 2014-04-14

**Authors:** Kyoko Ota, Mio Kawaguchi, Satoshi Matsukura, Masatsugu Kurokawa, Fumio Kokubu, Junichi Fujita, Yuko Morishima, Shau-Ku Huang, Yukio Ishii, Hiroaki Satoh, Nobuyuki Hizawa

**Affiliations:** ^1^Division of Clinical Medicine, Department of Respiratory Medicine, Faculty of Medicine, University of Tsukuba, 1-1-1 Tennoudai, Tsukuba, Ibaraki 305-8575, Japan; ^2^Department of Respiratory Medicine, Showa University Fujigaoka Hospital, 1-30 Aoba-ku, Yokohama 227-8501, Japan; ^3^Division of Allergy and Respiratory Medicine, Department of Internal Medicine, Showa University, School of Medicine, 1-5-8 Hatanodai, Shinagawa-ku, Tokyo 142-8666, Japan; ^4^Johns Hopkins University, Asthma and Allergy Center, 5501 Hopkins Bayview Circle, Baltimore, MD 21224-6801, USA; ^5^National Health Research Institutes, 35 Keyan Road, Zhunan, Miaoli County 35053, Taiwan

## Abstract

The expression of IL-17F is seen in the airway of asthmatics and its level is correlated with disease severity. Several studies have demonstrated that IL-17F plays a pivotal role in allergic airway inflammation and induces several asthma-related molecules such as CCL20. IL-17F-induced CCL20 may attract Th17 cells into the airway resulting in the recruitment of additional Th17 cells to enhance allergic airway inflammation. We have recently identified, for the first time, that bronchial epithelial cells are its novel cell source in response to IL-33 via ST2-ERK1/2-MSK1 signaling pathway. The receptor for IL-17F is the heterodimeric complex of IL-17RA and IL-17RC, and IL-17F activates many signaling pathways. In a case-control study of 867 unrelated Japanese subjects, a His161 to Arg161 (H161R) substitution in the third exon of the IL-17F gene was associated with asthma. In atopic patients with asthma, prebronchodilator baseline FEV1/FVC values showed a significant association with the H161R variant. Moreover, this variant is a natural antagonist for the wild-type IL-17F. Moreover, IL-17F is involved in airway remodeling and steroid resistance. Hence, IL-17F may play an orchestrating role in the pathogenesis of asthma and may provide a valuable therapeutic target for development of novel strategies.

## 1. Introduction

Asthma is characterized by bronchoconstriction, airway hyperreactivity, inflammation, mucus hypersecretion, and remodeling. These processes are coordinated by a complex cytokine network. The clarification of the modulation of this cytokine network could contribute to the understanding of asthma pathogenesis and development of new therapeutic strategies. IL-17A, the original member of the IL-17 cytokine family, was first identified in 1993 and was initially recognized for its similarity to a sequence belonging to the open reading frame 13 of* Herpesvirus saimiri *(HVS13) [1, 2]. Moreover, five additional members, IL-17B, IL-17C, IL-17D, IL-17E (also called IL-25), and IL-17F, were discovered within a short period of time since 2000 to 2002 [3–9]. Structurally, the IL-17 cytokine family members have no sequence similarity to any other known cytokine or other mammalian proteins [2]. Similarly, the IL-17 receptor family (IL-17RA-RE) is not related to any of the other known cytokine receptors [2]. Thus, the IL-17 cytokine family appears to represent a distinct ligand-receptor signaling system. We and other groups discovered the human IL-17F gene from a human EST sequence, a genomic DNA clone, and T-cell cDNA sequences in 2001 [3, 8, 9]. The gene is localized on the same chromosome at the distance of about 50 kb from telomeric sequences of IL-17A gene, and both genes are in a tail-to-tail orientation [3]. Functional studies have suggested that IL-17F is involved in asthma pathology. Hence, increased understanding of the significance of IL-17F would help to uncover the molecular mechanisms of asthma. In this review, we discuss the finding that IL-17F has a key role in asthma pathology and is a novel drug target for asthma.

## 2. Structural Features

Among the IL-17 cytokine family members, IL-17F shows the highest amino acid sequence homology (50%) to IL-17A, while only 10–30% sequence identity is seen between IL-17A and the other family members [10]. These cytokines have their greatest similarity within the C-terminal 70 amino acids and have four well-conserved cysteines. The four conserved cysteines in the C-terminal half of the IL-17F sequence are shown to form a cystine knot structural motif in the crystal structure, and, interestingly, this cystine knot structure is similar to a common structural motif seen in several growth factors, such as bone morphogenic proteins (BMPs), TGF-*β*, nerve growth factor (NGF), and platelet-derived growth factor (PDGF) [9]. Of note, recent reports have demonstrated that IL-17A and IL-17F can be produced as heterodimers termed IL-17A/F [11]. These three cytokines are differentially expressed in activated CD4^+^ T cells.

## 3. Cellular Source and Tissue Distribution

IL-17F is expressed in activated CD4^+^ T cells, basophils, and mast cells, three important cell types involved in allergic airway inflammation [3]. Moreover, IL-17F is also derived from Th17 cells, a CD4^+^ T-cell lineage distinct from Th1 cells and Th2 cells [12]. However, Th17 cells may not be the major cell source of IL-17F in lung diseases [13]. Recent studies have demonstrated that IL-17F is also produced by many cell types such as memory CD4^+^ T cells, CD8^+^ T cells, *γδ*T cells, NKT cells, B cells, and LTi cells [14–17]. These findings suggest that IL-17F is involved in the pathogenesis of a wide range of diseases beyond asthma. However, it remains to be determined which IL-17F-producing cell types contribute to asthma pathogenesis in response to various stimuli in human. So far, IL-17F has been thought to be derived from hematopoietic cells, but not nonhematopoietic cells such as lung structural cells. Recently, we have reported, for the first time, that bronchial epithelial cells are a novel cell source of IL-17F in response to IL-33 [18]. IL-33 is genetically and functionally associated with the pathogenesis of asthma [19, 20]. These findings suggest that bronchial epithelial cells play a central role in asthma, at least partially, as target and effector cells for IL-17F. In addition, IL-17F is detected in a wider range of tissues such as liver, lung, ovary, and fetal liver when compared with IL-17A [3]. This suggests that IL-17F has a more diverse biological function, despite the high degree of sequence homology with IL-17A.

## 4. Biological Activities 

**Figure 1:**
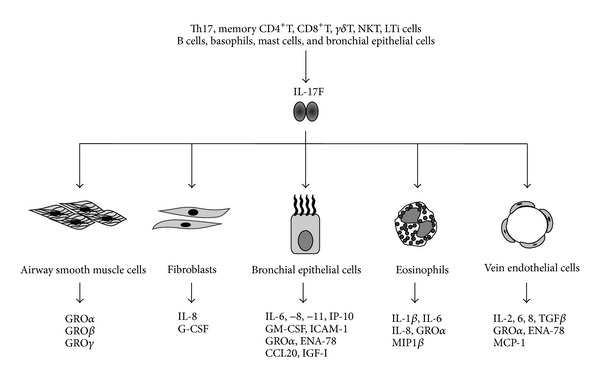
Biological activities of IL-17F. IL-17F has multiple biological activities. IL-17F is produced by several cell types including Th17 cells and bronchial epithelial cells. IL-17F can induce various asthma-related cytokines, chemokines, and adhesion molecules in bronchial epithelial cells, eosinophils, fibroblasts, airway smooth muscle cells, and vein endothelial cells, and thereby contributes to the pathogenesis of asthma.

IL-17F has multiple biological activities (Figure 1). IL-17F is able to induce asthma-related cytokines, chemokines, and adhesion molecules in bronchial epithelial cells [3, 20–28]. In addition to bronchial epithelial cells, IL-17F is also able to stimulate lung structural cells such as vein endothelial cells, airway smooth muscle cells, and fibroblasts [7, 8, 21, 22, 29]. Interestingly, a recent report demonstrated that IL-17F acts upon eosinophils, one of the most important inflammatory cells in allergic airway inflammation and remodeling, to induce several cytokines and chemokines such as IL-1*β*, IL-6, IL-8, GRO*α*, and MIP-1*β* [30]. These cell types may play crucial roles in asthma in response to IL-17F. IL-17F may develop and amplify allergic airway inflammation by facilitating the activation of inflammatory cells and lung structural cells through the induction of a wide range of molecules. Moreover, Th2 cytokines, IL-4 and IL-13, are able to enhance the biological activities of IL-17F [26–28, 31]. These findings suggest that the interaction of Th2 cytokines and IL-17F augments allergic airway inflammation.

## 5. Receptor and Signaling Pathway

**Figure 2:**
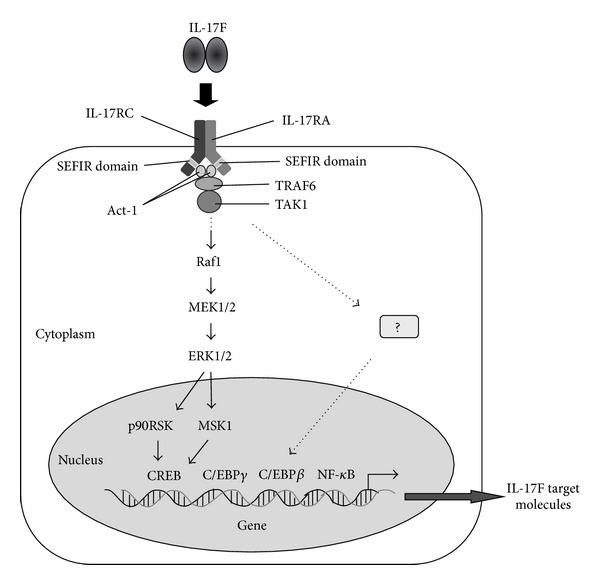
Signaling pathways induced by IL-17F. IL-17F utilizes a heterodimer of IL-17RA and IL-17RC as its receptor, and then IL-17RA engages the SEFIR domain-containing Act-1. Act-1 is required for recruitment of TRAF6, leading to the activation of TAK1. The Raf1-MEK1/2-ERK1/2-p90RSK/MSK1-CREB is the pivotal signaling pathway. On the other hand, IL-17F also activates transcriptional factors such as C/EBP*β*, C/EBP*γ*, and NF-*κ*B. Activation of these pathways leads to the expression of various inflammatory molecules.

**Figure 3:**
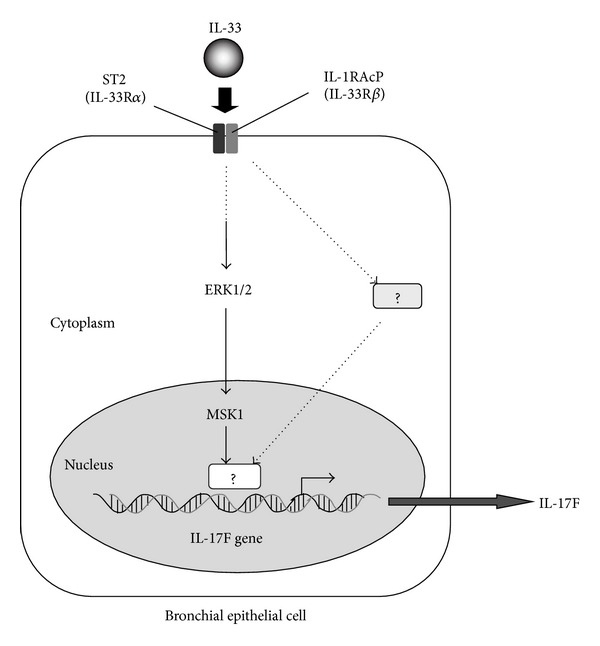
Signaling mechanism of IL-17F expression in bronchial epithelial cells. Bronchial epithelial cells are a novel cell source of IL-17F. The expression of IL-17F is induced by IL-33. IL-33 binds to its receptor, ST2, and then the ERK1/2-MSK1 signaling pathway is activated. However, other stimuli inducing IL-17F and their signaling pathways are largely unknown.

Our understanding of the signaling pathway of IL-17F has gradually become clearer (Figure 2). Similar to IL-17A, the receptor for IL-17F is the heterodimeric complex of IL-17RA and IL-17RC [32]. Both IL-17RA and IL-17RC are necessary for the biological activity of IL-17F. Although human IL-17RA binds IL-17A effectively, it binds IL-17F with ~1000-fold lower affinity [33]. The relative binding affinity of IL-17F to IL-17RC is much stronger than to IL-17RA. Activation of the receptor by IL-17F leads to an interaction with Act-1 via the similar expression to fibroblast growth factor genes, IL-17 receptors, and TIR (SEFIR) domain [34]. This sequentially mediates activation of TNF receptor-associated factor- (TRAF-) 6, leading to the activation of TGF*β* activated kinase (TAK) 1 [34, 35]. We have reported that the Raf1-MEK1/2-ERK1/2 pathway is a central upstream signaling pathway for IL-17F-induced cytokine and chemokine expression in bronchial epithelial cells and vein endothelial cells [21–28, 31]. In the downstream signaling pathway, we have also identified that mitogen- and stress-activated protein kinase1- (MSK1-) cyclic AMP response element binding protein (CREB) and p90 ribosomal S6 kinase- (p90RSK-) CREB are critical downstream signaling pathways [25–28, 31]. These pathways are located downstream of the Raf1-MEK1/2-ERK1/2 kinase cascade and are essential for cytokine expression by IL-17F. Further, IL-17F also activates transcriptional factors such as C/EBP*β*, C/EBP*γ*, and NF-*κ*B [34]. On the other hand, little is known about the signaling mechanisms of IL-17F expression. As shown in Figure 3, we have recently reported that bronchial epithelial cells are a novel cell source of IL-17F, and epithelial IL-17F expression is mediated via the activation of ST2-ERK1/2-MSK1 signaling pathway in response to IL-33 [18]. ST2 is a receptor for IL-33 [36]. However, other signaling molecules including transcriptional factors for IL-17F expression still remain undiscovered. These findings suggest that these signaling pathways are potential pharmacological targets in the IL-17F-mediated airway inflammation.

## 6. Recruitment of Th17 Cells 

Th17 cells play a pivotal role in a diverse group of immune-mediated diseases and host defense mechanisms [12]. Emerging evidence suggests that the Th17 cells provide a new insight into the molecular mechanisms of asthma [37]. Th17 cells have been isolated from bronchial tissues taken from patients during acute episodes of severe asthma [38]. Another study demonstrated that the percentages of Th17 cells in PBMCs are higher in allergic asthmatics than those in healthy subjects and show a tendency to increase with the disease severity [39]. However, it is unclear how Th17 cells migrate into the airway of asthmatics. We demonstrated that IL-17F induces CCL20 in bronchial epithelial cells [31]. Human Th17 cells predominantly express CCR6 [40]. This implies that its ligand, CCL20, is able to attract Th17 cells into the site of airway inflammation via CCR6. Taken together, it is possible that IL-17F-induced epithelial CCL20 attracts Th17 cells into the airway, and accumulated Th17 cells establish a positive feedback loop resulting in the recruitment of additional Th17 cells via the induction of IL-17F. Hence, IL-17F-producing cells may exert an effect on bronchial epithelial cells to induce CCL20 and attract Th17 cells via CCR6. Although* in vivo* study is needed to clarify this hypothesis, the IL-17F/CCL20 axis might be especially important in the pathophysiologic events of allergic airway inflammation.

## 7. Airway Neutrophilia 

IL-17F is involved in neutrophilic inflammation in the airway [41, 42]. Although airway neutrophilia is one of the hallmarks of severe asthma, its mechanism is not well understood. In a mouse model of study, overexpression of IL-17F using an adenoviral gene transfer strategy in the mouse airways also leads to an increased number of neutrophils in bronchoalveolar lavage fluid (BALF) [41]. Another study using a different model has revealed that overexpression of IL-17F through intratracheal delivery of the IL-17F gene results in an increase in the number of neutrophils and macrophages in the airways [42]. Moreover, IL-17F-deficient mice have revealed that IL-17F is more critical than IL-17A in inducing airway neutrophilic inflammation to* Aspergillus oryzae* [34]. Specific inhibition of CD4^+^ T cells, either with a CD4 Ab or an IL-2R Ab, prevents allergen-induced recruitment of both eosinophils and neutrophils in animal models, suggesting CD4^+^ T cells regulate airway neutrophilia [43, 44]. However, little is known about how CD4^+^ T cells elicit neutrophil accumulation into the airway. IL-17F may be one of the key regulators for airway neutrophilia induced by CD4^+^ T cells such as Th17 cells. Of interest is the finding that lung tissues from IL-17F gene transduced mice show substantial increases in the level of various inflammatory cytokines and chemokines, including IL-1*β*, IL-6, KC, and MIP-2 [41, 42]. These molecules are known to be involved in chemotaxis and activation for neutrophils. Additionally,* in vitro* studies have demonstrated that IL-17F is able to induce C-X-C chemokines, such as IL-8, ENA-78, and GRO*α*, which are potent chemoattractants for neutrophils [3, 21, 22]. Neutrophil recruitment into the airway may be regulated through, at least partially, IL-17F-induced C-X-C chemokines. In contrast, C-C chemokines, such as eotaxin and RANTES, which are potent chemoattractants for eosinophils, are not produced by IL-17F, suggesting a selective role of IL-17F in neutrophil recruitment and activation in the airway [3].

## 8. Mucus Hypersecration and Airway Hyperreactivity

Asthma is characterized by mucus hypersecretion (goblet cell hyperplasia/metaplasia) and airway hyperreactivity that are consistently linked to asthma symptoms and morbidity. IL-17F may be involved in these pathological processes. Overexpression of IL-17F in the airway of mice resulted in the induction of goblet cell hyperplasia and the gene expression of MUC5AC, but only when the mice are challenged with antigen, and increased goblet cell hyperplasia is seen only in the small airways [42]. These results suggest that in addition to IL-13, IL-17F may also be an important contributor to mucus hypersecretion in asthma. Moreover, a significant increase in airway hyperreactivity was also noted in mice overexpressing IL-17F following Ag challenge, when compared to that of mice receiving mock control [42]. These findings suggest that IL-17F has an additive or enhancing effect on antigen-induced allergic inflammatory responses.

## 9. Airway Remodeling

We have reported that IL-17F induces profibrotic cytokines, IL-11 and IGF-I, in bronchial epithelial cells [27, 28]. IL-11 elicits subepithelial fibrosis, accumulation of fibroblasts, myofibroblasts and myocytes, and deposition of types I and III collagen [45]. IGF-I is able to induce collagen synthesis and smooth muscle hyperplasia and is also a potent mitogen for fibroblasts and smooth muscle cells [46–48]. The blockade of IGF-I inhibited the elevation of airway resistance, airway inflammation, increase in airway wall thickening, and the expression of ICAM-1. In humans, the expression of IGF-I is significantly increased within the airways of subjects with severe asthma when compared with those with mild asthma [49]. Its expression was inversely correlated to collagen thickening and the number of fibroblasts. Moreover, treatment with beclomethasone dipropionate significantly decreased the expression of IGF-I with reduction of the thickness of lamina reticularis. Blocking of IGF-I expression may contribute to prevent airway remodeling. In other studies, IL-17F has been shown to induce the expression of TGF-*β* in vein endothelial cells [8]. TGF-*β* is a profibrotic cytokine and has been implicated in the extracellular matrix changes observed in fibrosis. More recently, direct effect of IL-17F to airway smooth muscle (ASM) cells was demonstrated. IL-17F promotes migration of ASM cells via p38MAPK [50]. Th17 cells contribute to airway remodeling via excessive mucus expression and ASM proliferation [51]. These findings suggest the potential involvement of IL-17F in the process of airway remodeling.

## 10. Steroid Resistance

Recent studies have demonstrated that IL-17F is involved in steroid resistance in asthma. Th17 cells, but not Th2 cells, mediate steroid resistant airway inflammation and airway hyperreactivity in a mouse model of asthma [52]. In the setting of* in vivo* polarized Th17 cell transfer, chemokine secretion, cellular influx to the airways, and airway hyperreactivity are not sensitive to dexamethasone treatment. Other studies have reported that IL-17F induced expression of glucocorticoid receptor- (GR-) *β* mRNA in bronchial epithelial cells from healthy subjects as well as asthmatic patients [53]. GR-*β* acts as a dominant negative inhibitor of GR-*α* which is the active isoform of this receptor. Moreover, unlike healthy subjects, IL-6 induced by IL-17A and IL-17F was not inhibited by dexamethasone in bronchial epithelial cells from asthmatic patients. These findings suggest that steroid resistance in subjects with severe asthma may be due to, at least in part, IL-17F and Th17 cells.

## 11. Expression in the Airway of Asthmatic Patients

The expression of IL-17F is observed in the airway of asthmatic patients. Analyses of its expression in BAL cells from asthmatic subjects challenged with allergen or saline control show that while no detectable expression of IL-17F was seen in the BAL cells from saline-challenged sites, its expression was obviously seen in the BAL cells from allergen-challenged sites of all four study subjects [3]. IL-17F is expressed in both bronchial epithelium and inflammatory infiltrates [54, 55]. Immunocytochemistry showed that IL-17F positive cells in the subepithelial component and epithelium are significantly elevated in severe asthma compared with healthy and mild asthmatic subjects [55]. Additionally, an increased expression of epithelial IL-17F was correlated with disease severity. Moreover, another recent study demonstrated that asthmatic patients have a significantly higher level of serum IL-17F protein as compared to that of healthy subjects [56]. This implies that IL-17F can be used as a clinical biomarker of asthma diagnosis and management. Further validation is needed in the future.

## 12. Genetic Relevance 

We investigated the genetic association of asthma with the common variants of IL-17F, using 867 unrelated Japanese subjects [57]. Five polymorphisms were studied, including the coding-region sequence variant SNP rs763780 (7488T>C), which causes a His-to-Arg substitution at amino acid 161 (H161R). A genotype-based *χ* ^2^ association analysis indicated a significant association between the H161R variant and asthma. Importantly, none of the asthmatic subjects were homozygous for H161R. The homozygosity of the H161R variant is associated with the protection against asthma; the odds ratio (OR) for asthma was 0.06 (95% confidence interval, 0.01–0.43, *P* = 0.0039) among H161R homozygotes compared with wild-type homozygotes. In atopic patients with asthma, prebronchodilator baseline FEV1/forced vital capacity (FVC) values also showed a significant association with the H161R variant [58]. This suggests that the H161R variant of IL-17f is associated with asthma severity. Moreover,* in vitro* functional studies demonstrated that, compared with wild-type IL-17F, the H161R variant is unable to activate ERK1/2 that is a critical signaling molecule of IL-17F [57] but, interestingly, is able to block the induction of IL-8 by wild-type IL-17F in a dose-dependent manner. These findings suggest that the H161R variant is a natural antagonist for the wild-type IL-17F and may be an attractive therapeutic target in IL-17F-mediated diseases. However, further study is needed to clarify the precise mechanisms by which H161R variant exerts its antagonistic effect. Interestingly, recent studies have demonstrated novel therapeutic options targeting IL-17A, IL-17F, and their signaling pathways. Inhibition of either IL-17RA or IL-17RC expression via siRNA revealed significant reduction of IL-17A/IL-17F-stimulated chemokine production [59]. Similarly the microRNA, miR-23b, suppresses IL-17A-associated autoimmune inflammation by targeting TGF-*β*-activated kinase 1/MAP3 K7 binding protein (TAB)2, TAB3, and IKK-*α* [60]. These molecules may provide therapeutic benefit for immune and inflammatory diseases.

## 13. Conclusions

IL-17F is one of the important cytokines involving in the pathophysiologic events of asthma.* In vivo* and* in vitro* studies have implicated that IL-17F shows multiple functions in the pathogenesis of airway allergic inflammation. In particular, CCL20 induced by IL-17F may enhance Th17-mediated airway inflammation via CCR6. Although biological function of IL-17F has become clear, its inducible factors still remain except for IL-33. It is suggested that further investigation of IL-17F is informative in pointing to novel approaches to the diagnosis and treatment of asthma.
